# Still engaged – healthcare staff’s engagement when introducing a new eHealth solution for wound management: a qualitative study

**DOI:** 10.1186/s12913-022-07515-3

**Published:** 2022-01-25

**Authors:** Cecilia Fagerström, Hanna Wickström, Hanna Tuvesson

**Affiliations:** 1 Faculty of Health and Life Sciences, Linnaeus University, SE-391 82 Kalmar, Sweden; 2 The Research Section, Kalmar, Region Kalmar County Sweden; 3 Department of Clinical Sciences, Malmö, Center for Primary Health Care Research, Lund University, Malmö, Sweden; 4 Blekinge Wound Healing Centre, Karlshamn, Sweden; 5 Faculty of Health and Life Sciences, Linnaeus University, Växjö, Sweden

**Keywords:** Engagement, Hard-to-heal ulcers, Decision support, eHealth

## Abstract

**Background:**

eHealth solutions have often been considered favourable for improved effectiveness and quality in healthcare services for wound management. Staff engagement related to organisational changes is a key factor for successful development and implementation of a new eHealth solution, like a digital decision support systems (DDSS). It is essential to understand the engagement process in terms of sustainability, wellbeing in staff and efficiency in a long-term perspective. The aim of this study was to describe healthcare staff’s engagement during a 6-month test of an eHealth solution (DDSS) for wound management.

**Methods:**

A qualitative design, including interviews conducted with healthcare staff working with wound management within primary, community and specialist care (*n* = 11) on two occasions: at the introduction of the solution and after 6 months, when the test period was over. Data were interpreted with qualitative content analysis.

**Results:**

Healthcare staff’s descriptions from a 6-month test of an eHealth solution for wound management can be summarised as *Engaging through meaning, but draining*. The analysis revealed a result with three subcategories: *Having a shared interest is stimulating, Good but not perfect* and *Exciting, but sometimes exhausting.* The staff described their engagement as sustained through feelings of meaningfulness when using the eHealth solution, but limited by feelings of exhaustion due to heavy workload and lack of support and understanding from others.

**Conclusions:**

The results indicate that the healthcare staff who tested the eHealth solution described themselves as individuals who easily become engaged when an idea and efforts felt meaningful. The staff needed resources to nourish engagement in their new role when implementing eHealth in the clinical everyday work of wound management. Allocating time and support are important to consider when planning for sustainable implementation of eHealth solutions in healthcare organisations.

## Background

At a time when more and more healthcare is moving into patients’ homes, where medical expertise is not always available, it is becoming increasingly important to emphasise management support and cooperation between care units. Treatment of hard-to-heal ulcers (ulcers which take more than 4–6 weeks to heal) [1, 2] is mainly performed in the home care organisation and at primary care centres and is demanding and time-consuming for both patients and staff [3]. The costs of wound management are difficult to estimate, but most are related to healthcare staff’s time and extended hospital stays, and the economic impact on the healthcare system is significant [4]. Wound management is frequently delivered by nurses, independently or together with nurse assistants, in a context where the staff often works without any structural guidelines, with insufficient medical support [5, 6] and the knowledge about wound management is limited [6]. The healing time can extend from several months to years. Successful collaboration across organisational boundaries is fundamental for treatment, diagnosis and follow-up in wound management [7]. eHealth solutions are expected to have high potential to improve the working conditions, especially in home care settings [8].

eHealth refers to usage of information and communication technologies for health [9] and encompasses various forms of digital transmission of imaging and clinical data. Telemedicine is one type of eHealth solution that enables medical care [10] and has been introduced into healthcare to increase accessibility and facilitate care [11, 12]. A literature review has shown that there is strong evidence that eHealth solutions in wound management have high quality and are clinically effective, but limited evidence on ethical aspects, which could be used to identify potential discomfort for the end users when they use the solutions [13]. Telemedicine and eHealth solutions in wound management generally show positive outcomes in terms of staff satisfaction [13], improved communication [14, 15] and ulcer pain assessment and treatment [11], reduced healing and waiting time for patients [12], increased access to expert advice outside hospitals [5], improved knowledge [5] and improved skills and heightened confidence [14, 15]. Furthermore, one of the studies reported [15] that eHealth solutions made it possible for staff to understand their patients in a more holistic way.

One commonly used example of an eHealth solution is the digital decision support system (DDSS), which serves to support medical decisions and education in everyday clinical practice [16]. Such systems are getting increased attention because they can promote collaboration and cooperation between different healthcare professions [17], something that is particularly important for patients with hard-to-heal ulcers. A DDSS can be used in home care by a nurse or nurse assistant visiting the patient, to enable provision of expert advice from wound management teams in primary or specialist care without the patient being obliged to travel to a clinic.

Engagement is often described as crucial for the introduction [18] of new eHealth solutions. The process of engagement is multidimensional, complex and involves cognitive, emotional and behavioural factors [18]. Engagement in eHealth contexts has been described in various ways, from a psychological process related to user experiences and perceptions to a general view of usage of an intervention [18–20]. Commonly, engagement is described in terms of the extent, frequency, duration and depth of usage [18]. Attitudes connected to engagement as a cognitive dimension and clinical engagement behaviour to develop patient safety and quality of care are also mentioned in the literature [21]. Furthermore, engagement at the personal level depends on active support from the surroundings and individual experiences such as relevance, interest and enjoyment [17].

Although many eHealth solutions have been introduced and evaluated, staff engagement process during introduction of eHealth solutions in wound management is poorly understood. Combining already established structures and clinical practices with new eHealth solutions may be challenging, and both staff and patients need time to adapt and learn to use any new solution successfully [8]. In wound management, which is already demanding, engagement in a new work process like using an eHealth solution may lead to increased demands as well as new roles and clinical tasks, often negatively associated with increased moral distress [22, 23]. The engagement process needs to be investigated more deeply from a long-term perspective, in order to improve the possibilities for successful implementation and prevent increased unhealthy workload and stress.

In summary, although eHealth solutions are used in modern wound management, no studies have focused on the engagement process related to eHealth interventions, such as a DDSS in wound management. Evaluation of staff engagement in using a DDSS has previously been described in the early stage when the solution was new and unfamiliar to the users (cf. [17]). However, it is also essential to understand the engagement process in the long term, as regards sustainability, staff wellbeing and efficiency. Thus, the aim of this study was to describe healthcare staff’s engagement during a 6-month test of an eHealth solution for wound management. The following questions were addressed:

- How do healthcare staff describe their engagement over time?
- Does the healthcare staff’s engagement change over time and if so how and why?
- What are the potential reasons for maintaining/not maintaining engagement over time?

## Methods

A qualitative design was chosen for this study. Qualitative methods facilitate in-depth study of a topic through openness to data and individuals’ descriptions of the situation under study [24]. Individual interviews were conducted in order to capture the healthcare staff’s experiences of engagement when being involved in a DDSS intervention for wound management. Conventional content analysis was used to interpret the text data from a naturalistic paradigm [25], conceptualised by the healthcare staff’s descriptions of engagement in their context, independent of frequency of usage.

### Settings

In Sweden, most patients with hard-to-heal ulcers are treated in primary care and community care [6], where healthcare staff – often nurse assistants, nurses and physicians – cooperate in wound management teams. Specialist clinics get involved after referral for specific treatment, such as revascularisation or venous surgery.

Dermicus Wound, the DDSS used in present study, was developed by a Swedish company, in collaboration with the Swedish national quality registry, the Registry of Ulcer Treatment (RUT) (https://www.rikssar.se/forskning-english/). Dermicus Wound includes a mobile application for bedside registration in RUT and transfer of medical data and images to a web browser-based platform for multidisciplinary communication and consultation. The DDSS is intended for use by multiple healthcare professionals, supporting them with structured processes for clinical teams to improve wound management: diagnosing and treating ulcers and reducing healing time and patient suffering. For more information, see previous publications [17]. Dermicus Wound is CE-certified as a Medical Device, Class I (D3.0–112,015), and compatible with iPhone devices, using a standard touchscreen user interface and camera.

### Participants

Dermicus Wound was deployed in 2018, when a trial version was made available for testing for free among registrars in RUT. The staff was recruited at an annual user meeting and via RUT’s website, where information about the study was provided by the company behind the DDSS and by the chairman of RUT’s steering group. In total, 65 healthcare workers agreed to test and evaluate the DDSS for 6 months and give their opinions about it. From this group, 11 volunteered and consented to be interviewed about their engagement in the DDSS. The participants were general practitioners (*n* = 2), nurses (*n* = 7) and nurse assistants (n = 2) frequently treating patients with hard-to-heal ulcers. Eight participants were women and three were men. All participants got the same information and technical support, and were invited to contribute with improvement suggestions during the test period.

### Data collection

The 11 participants used the DDSS during a 6-month test period and were interviewed on two occasions each, answering questions about their engagement in the DDSS. The first interview was conducted within the first month after starting to use the DDSS, and the second, i.e., the follow-up interview, after the 6-month test period. This study was carried out between October 2018 and October 2019. An interview guide, encompassing questions regarding engagement during the testing of the DDSS and reasons for maintaining/not maintaining engagement during testing, was used. All interview questions were open-ended. The same interview guide was used in both interviews, but the time frame was changed from 1 month to 6 months for the follow-up interviews, to capture descriptions over time. In the first interviews, a question about individual engagement in relation to new work processes was also used, to introduce the participants to the field. The interviews were recorded and transcribed verbatim. The length of the interviews at start-up varied from 29 to 48 min and the interviews at end of the test period were between 21 and 45 min long.

Before the interviews were carried out, the participants were informed verbally and in writing about the study, confidentiality and the voluntary nature of participation. All participants gave written consent. The project was approved by the Ethical Advisory Board in southeast Sweden (registration number EPK 506–2018). All methods were carried out in accordance with national guidelines and regulations. The interviews were conducted by all three researchers (HW, HT, CF) by turns. The researchers’ pre-understanding was based on the experiences of being a specialist in wound management (HW) and ongoing research in the engagement process (CF, HT, HW).

### Data analysis

Data were interpreted with qualitative content analysis in accordance with the inductive approach of the model developed by Elo and Kyngäs [26]. The model corresponds to conventional content analysis [25]. The researchers each individually read all 22 (11 + 11) transcribed interviews and field notes. With the aim of the study in mind, notes were written in the margin of the interview texts and field notes to organise the data [25]. These notes were then shared among the researchers. The text was divided into units of meaning, sentences, and paragraphs, and then condensed, without removing their core. Codes were identified in the condensed text; these were compared with the original transcribed texts and field notes to ascertain that the context was maintained by the codes. A coding scheme was created in which codes were assigned different colours depending on if they resulted from a first interview or a follow-up interview. The codes were then compared and classified into groups or subcategories. Similar subcategories were gathered in main categories to provide a means for describing the phenomenon. The analysis went back and forth from text to categories through a systematic classification process, with potential differences in nuances between the first interviews and the follow-up interviews taken into consideration. To increase validity, all of the researchers analysed the text separately and then compared and discussed their listed units of meaning, codes, subcategories and categories. Lastly, all researchers re-read the transcribed interviews to ensure that the main category and subcategories as well as the relationships between them reflected what the staff described in the interviews [26].

## Results

The analysis of the healthcare staff’s descriptions of engagement during a 6-month test of an eHealth solution for wound management revealed one main category: *Engaging through meaning, but draining*. The main category was described through three subcategories: *Having a shared interest is stimulating, Good, but not perfect* and *Exciting, but exhausting.*

### Engaging through meaning, but draining

Healthcare staff’s engagement during a 6-month test of an eHealth solution for wound management can be described as being *Engaging through meaning, but draining*. A sense of meaningfulness was experienced when engagement was shared, when feelings of purpose and improvement of care were maintained and when staff members were given room to become excited. When experiencing a lack of these aspects, feelings of exhaustion left many staff members feeling drained, with resulting health consequences. Still, their personal engagement in the patient group and eagerness to help them was described as being preserved, surviving many obstacles and shortcomings.

#### Having a shared interest is stimulating

Having a shared interest in the project of wound management and the eHealth solution was described as a key for triggering initial engagement, nurturing engagement over time and overcoming periods of tiredness related to work environment obstacles. Sharing an interest stimulated feelings of meaningfulness, which built engagement throughout the test period. When interest was not shared, some staff members felt forced to end their involvement during the test period.

At the beginning of the test period, healthcare staff stated that their engagement was set into motion through external colleagues’ spark and enthusiasm for the project and/or hard-to-heal ulcers. Experiencing a ‘kick up the backside’ from colleagues, the project team or managers led to an increase in own engagement. This initial spark was also described as quickly fading upon return to everyday work life without these external sources of inspiration. Over time, having someone else who moved things forward was described as important for maintaining engagement throughout the test period for some of the staff. Others tried to be the engine driving the project forward themselves and stimulate the shared interest.

*… it is incredibly inspiring and we all feel that way in the group when we have been on these user days. Then we come home and are filled with energy and feel like we are going to save the world, but then that doesn’t quite happen … (Start-up interview no 5)*

Sharing an interest with colleagues felt stimulating, which created joy and excitement. The engagement of others was experienced as ‘infectious’ and stimulating own engagement. For those who had not used the eHealth solution during the test period, a feeling of being alone, without supporting and encouraging colleagues, was described as wearisome and an obstacle for sustained engagement. A shared understanding of the potential usefulness of the eHealth solution in wound management was considered to be important for shared engagement in the group.

*… if I was alone and didn’t, like, get anyone to join me, then it isn’t any fun either. You kind of need some encouragement from the others and, like, to feel that what you are doing seems meaningful … (Start-up interview no 1)*

Having a leader who showed the way and prioritised the project was considered to be fundamental for shared interest and for stimulating and maintaining engagement during the test period. For healthcare staff who had not used the eHealth solution during the test period, the lack of leader engagement was expressed as a major barrier to engagement, sometimes creating a sense of draining and lack of meaning. Some leaders were described as not understanding the value of the project and prioritising other things. A change in leadership could mean that the leader engagement changed from full support at the beginning to direct orders to terminate participation later during the test period.

*… there’s a lack of support from our managers … [our former boss] was awesome and supported us so much in this … but she’s no longer working here … we all miss using the app and thought it was great … it’s a shame we can’t use it anymore … (Follow-up interview no 4)*

#### Good, but not perfect

Initial engagement was described as being strongly connected to needs and benefits related to work, care and the patients. When the need for change or improvement came from within, from the staff or from patients and their families, it felt meaningful – which triggered and nurtured engagement during the test period. Some participants expressed initial scepticism towards eHealth in general and thought it should not simply assumed to be helpful, but had to be justified. Initial criticism was expressed when the purpose of the eHealth solution was not clearly communicated to and understood by the staff. Towards the end of the test period, new thoughts appeared in relation to what would happen next. It was not clear if they would be allowed to continue to use the solution or if there was any long-term plan for implementation. This uncertainty and lack of predictability influenced the sense of meaningfulness in use of the solution, resulting in feelings of weariness which affected engagement detrimentally.

*… because it is always like that with projects … so that it can live on, because that is the kind of thing that can make me lose engagement sometimes, because, well, but why should I do it, if there is no continuation … (Follow-up interview no 3)*

The healthcare staff expressed a great need for improving wound management and helping the patient group in a better way. They longed, wished and hoped for more structure, continuity and safety in the care processes and management, which they expected the eHealth solution to bring. At the end of the 6-month test period, expectations and wishes were often met. The solution improved efficacy and quality by for example reducing unnecessary travel for patients and staff, promoting more thorough and structured examinations and providing expert advice already in home care and primary care.

*It’s a huge advantage that you can choose to look at the images when you have the time … you don’t need to call someone and interrupt that person’s work, or necessarily bring a physician to the room … I can wait with responding … when it’s a better time … it makes work a lot easier. (Follow-up interview no 9)*

For many participants, use of the solution had become a routine which felt simple and natural and which was missed when they had to terminate use at the end of the test period. The improvements were described as promoting work satisfaction and engagement. Still, the need for technology- and content-related improvements was strong. The solution was not as easy to use as the staff would have wanted, and they felt that it should have been more ‘finished’ and complete prior to testing. Some felt that the technological shortcomings created fatigue and drained them of energy. Desired functions failed to materialise. For some, a lack of quick access to patient information lead to worries regarding patient safety and an increased workload as they had to check existing documentation.

*I’ve noticed … that I don’t remember … like, you miss a lot of this, well … this wound has been here for four weeks … I miss, like, how did it arise … now maybe I have to access the medical records to remind myself of that … (Follow-up interview no 3)*

#### Exciting, but exhausting

Being involved in the testing of an eHealth solution influenced the healthcare staff personally in various ways. During the 6-month test period, engagement was characterised by ups and downs and a mix of feelings such as interest, joy and excitement, but also stress, disappointment and exhaustion. For some, the test period involved continuous use of the eHealth solution, with a lasting desire to move forward and improve wound management. Though engagement was described as being more effervescent and energetic initially, it was maintained with a strong focus over time. Many staff members found that their personal engagement outlived challenges, but for others insufficient resources led to exhaustion and the termination of testing to protect their health. Engagement was described as remaining, but being drained. They described optimistic visions of engagement in the use of the eHealth solution returning if conditions were to improve.

*The engagement is there and the ambition to get it to work, but as I said … there’s not enough time to learn about it and see what you need it for, and sometimes you have to put something on the back burner so you don’t … it can use up so much energy that you won’t have any left for something else (Follow-up interview no 4)*

The healthcare staff described themselves as driving forces who easily became engaged and wanted to do things well, up to a high standard. They worked overtime, skipped lunch and made an extra effort in testing the solution. Receiving affirmation for their efforts made the work feel engaging, fun and meaningful. However, the strain of this and deficits in the work environment and technology during the introduction left many stressed, exhausted and feeling like failures. Technological shortcomings and complications, combined with having no time allocated for the testing and introduction, created stress, irritation and a feeling of not being able to do what they had set out to do. During the testing period, this led to sadness and fatigue for several staff members.

*When we used it, I felt very engaged … and I kind of invested time in it and even time beyond my regular working hours, because you thought it was so much fun and important and so … I remember that, feeling very engaged back then … Now we have a rather changed work situation here … everything has kind of been put on hold … so you lose engagement for your work in general … you can’t … then you could risk your health … (Follow-up interview no 5)*

Some participants perceived the conditions during the test period as appropriate. They adapted to technological shortcomings and strived to gain control and use the possibility to change circumstances in health care. The staff felt valued and saw themselves as explorers, whose opinions were considered important, which stimulated engagement. They felt excited to participate in the development process. Use of the eHealth solution led to extra work, but was perceived to be easy for those who had the time to properly familiarise themselves with the system.

*… I understand that development takes time … This is the future in some sense, I guess I’ve been waiting for something like this for many years. (Follow-up interview no 1)*

## Discussion

eHealth solutions have often been considered favourable for improved effectiveness and quality in health care services. Engagement connected to organisational changes due to new eHealth solutions is a key factor for successful development and implementation of such solutions [20]. However, the experiences and processes of engagement are complex and there is a need for a more holistic understanding thereof. For these reasons, we decided to target healthcare staff’s descriptions of engagement during a 6-month test of an eHealth solution for wound management. The healthcare staff described their engagement in the eHealth solution as being linked to a feeling of meaningfulness and high eagerness to improve the care of patients with hard-to-heal ulcers. This came with exhaustion, fatigue and feelings of being drained if proper organisational resources were lacking or the solution did not meet initial expectations for improvements, hindering successful implementation.

A shared understanding of the potential usefulness of the eHealth solution in wound management and engagement among others in the group, including stimulating and satisfactory leadership, were described as important for engagement over time. Collegial and organisational support being described as valuable in building positive experiences in implementation of eHealth solutions is supported by experiences from the introduction of the DDSS [17] and findings from a review conducted by Konttila and colleagues [27]. Kahn [28] also suggested that social connections, such as work interactions and interpersonal relationships, are crucial sources of meaning and that work climates characterised by openness and support help people dare to ‘risk’ being engaged. Further, the findings from the present study revealed that the social influence connected to the staff’s engagement during the test period changed over time. When the project team or managers grew less visible than during the introduction, the participants themselves or together with close colleagues took over as the driving and supportive forces, to stimulate engagement and move the project forward. With the value of the project in mind – based on needs and benefits for work, care and patients – the project participants’ interactions continued. They strived to do what they had set out to achieve during the test period. The influence of positive sustainable collegial support and a positive social environment at the workplace was highly important to maintain engagement in the eHealth solution over time. This carries its own challenges, as we know from the literature that eHealth solutions affect the psychosocial atmosphere both positively and negatively in everyday clinical practice [27, 29, 30].

The association between engagement and meaningfulness has been described by Kahn [28], who found that people tend to have stronger personal engagement when perceiving a situation as more meaningful. According to Kahn [28], people experience meaningfulness when they feel useful and valuable and sense that their efforts have made a difference. Similar aspects were described by the healthcare staff in the present study, for example when they felt their opinions were valued and they were part of the process of improving care for patients with hard-to-heal ulcers. In a similar vein, Keyworth et al. [31] argued that interventions in eHealth should take into account organisational settings and clinical workload and clearly indicate how they can improve clinical practices and patient outcomes, in order to create sustainable engagement among staff. eHealth should support professionals’ work, expand their capacities and provide opportunities to add value [29]. Otherwise, sustaining engagement with strong emotions can be demanding, especially when there is a lack of meaning, as described in the present study.

In the present study, engagement begin affected by feelings of being drained should be understood in relation to the healthcare staff’s descriptions of fatigue and exhaustion. Staff members sometimes needed to take action and prioritise clinical work and other tasks not related to testing of the new eHealth solution. Increases in workload and stress when implementing an eHealth solution for patient self-management have previously been acknowledged among primary healthcare nurses [32]. It is known that adoption and development of eHealth solutions rely heavily on the staff involved [33, 34] and their previous experiences of eHealth. Technological shortcomings and solutions that are not adapted for the users or the specific context increase stress levels in healthcare staff [35]. Hence, signs of emotional exhaustion connected to new eHealth solutions, potentially having consequences for staff health, need to be highlighted.

Furthermore, the findings indicated that not being engaged, through not actively participating in the testing, might have been a personal strategy for staff members to protect their health. Although this was not investigated in this study, it can be a way to reduce the risk of overwhelming fatigue, which is described as one of three key dimensions in burnout [36]. However, non-participation also affected wellbeing negatively, as staff knew that the project had a good purpose. The participants described a sense of insufficiency and unavailability in relation to the project team, colleagues and patients. Thus, it is important to find ways to prevent a lost sense of wellbeing in eHealth initiatives. Otherwise, there is a risk that work will be prioritised based on prevailing work conditions at the individual and organisational level, potentially leading to lower engagement, even in a worthwhile new eHealth initiative. Experiences of simplicity, agility and immediate usability can therefore have a crucial impact on whether or not staff will adopt and use new technology. Using an approach of person-centred design to better meet staff needs and achieve acceptance seems crucial [34].

Based on Konttila et al. [27], the discussion of Kho et al. [37] and the results of the present study, one could argue that the response from the participants would be more positive if they had been involved in the development of the eHealth solution. The results indicated that being part of the implementation or promotion of digital health services was related to significantly more positive experiences of engagement during the test period and attitudes towards the eHealth solution. Furthermore, including trial participants in the development of eHealth solutions, to capture existing knowledge on practical issues, routines and obstacles in daily clinical practices, has been proven to be helpful in reducing the risk of low activity and low participation in trials [38]. In the early introduction of the present DDSS [17], having time for adjustment and learning was emphasised as important when the staff described engagement, due to varied levels of knowledge, previous experience and interest in eHealth solutions. For sustainable engagement and implementation, staff need time to learn how to use a new solution and create new routines while also carrying out their everyday clinical work [17]. They need resources for new roles and adaption of the new organisational work process [39]. Allocating time for clinical staff to do this and to assimilate their new role can be considered to be important when planning the implementation of eHealth solutions, in order to promote wellbeing and ensure that the staff has the energy to interact in the development process without growing exhausted.

### Strengths and limitations

The participants were representative of all the different disciplines involved in wound management teams (physicians, nurses, and nurse assistants), and came from community, primary and specialist care. In addition, the participants were from different parts of Sweden, both rural and urban areas, and included both females and males, which is important for the transferability of the findings [40]. All participants had experiences of the phenomenon under study, ensuring credibility [40]. To enable readers to judge the credibility of the results, and to prioritise the voices of the participants, citations were used [40]. Further, each interview was briefly summarised at the end of the interview session, to allow the participant to confirm if the researchers had understood them correctly. Triangulation was used between the three moderators, increasing credibility. One possible limitation is that the participants volunteered to participate, creating a selection likely to have positive attitudes towards eHealth interventions. Another possible limitation was created by the practical constraints in terms of the limited number of potential participants that could be recruited and included in the study, which may have prevented saturation from being reached. We included all participants who volunteered, but were unable to recruit additional participants for the second interview due to the follow-up approach. However, in the present study, saturation was assessed in relation to the richness of the data and identification of new codes in the analysis, and we deemed appropriate for establishing data quality. To increase dependability, all three moderators were involved in the analysis [41], and their preunderstanding was highlighted and described [24, 26, 40]. In addition, memos were used to keep track of changes during the process. A strength is that none of the moderators were involved in using or deploying the eHealth solution, improving confirmability [42].

## Conclusions

The results indicated that the healthcare staff who agreed to participate in the testing of the eHealth solution described themselves as individuals who easily become engaged in ideas and efforts that felt meaningful. When staff members were engaged, they were passionate and pushed themselves hard in order to do what they had set out to do, both in the project and in their clinical work. At the same time, these personal attributes in many cases contributed to feelings of being drained and exhausted when not having access to the proper resources or when the eHealth solution did not meet initial expectations. Thus, when planning for sustainable implementation of eHealth solutions in healthcare organisations, it is important to take account of management support and allocation of sufficient time to give staff a chance to grow familiar with the solutions and any new tasks.

This study focused on engagement in staff over time. Based on the result that staff engagement when introducing an eHealth solution was affected by stress, disappointment and exhaustion, it is important to further deepen our knowledge about the links between implementation of eHealth solutions, work environment and the health situation among staff.

## Abbreviations

DDSS: Digital decision support system
RUT: Registry of Ulcer Treatment
